# Supplementary material for: An interactive meta-analysis of MRI biomarkers of myelin
Source: eLife. 2020 Oct 21;9:e61523. doi: 10.7554/eLife.61523 (PMC7647401; doi:10.7554/eLife.61523)
Supplement: Supplementary file 1. [file elife-61523-supp1.zip › interactive_figures.html]

interactive\_figures\_rev1
